# Emotional analysis of evaluation discourse in business English translation based on language big data mining of public health environment

**DOI:** 10.3389/fpubh.2022.981182

**Published:** 2022-10-20

**Authors:** Song Liu, Yukun Chen, Kunpei Xu, Jiaxin Lin

**Affiliations:** ^1^School of Foreign Languages, Hunan University of Finance and Economics, Changsha, China; ^2^School of Humanities, Nanyang Technological University, Singapore, Singapore; ^3^School of Foreign Languages, East China Normal University, Shanghai, China; ^4^School of Foreign Studies, Northwestern Polytechnical University, Xi'an, China

**Keywords:** language big data mining, public health environment, clustering algorithm, business English translation, discourse emotion

## Abstract

**Purpose:**

This paper conducts sentiment analysis on the evaluation discourse of business English translation based on language big data mining of public health environment, and aims to find a reasonable algorithm to conduct detailed research on all aspects of sentiment analysis.

**Methodology:**

This paper focuses on three areas of sentiment information, extraction, sentiment information retrieval, and sentiment information submission, using scale analysis and feedback analysis, combined with related algorithms of big data mining technology, such as decision trees and clustering algorithms, through the level of emotional words appearing in the corpus, phrase-level, text-level, etc., and combine the text model with the combined reliability to output the evaluation object and evaluation feature separately, and propose an evaluation method to calculate the sensitivity of the evaluation feature, so as to accurately improve the sensitivity of the evaluation feature. It is mainly divided into two categories for data analysis. One is to focus on the public health environment of the characteristics of business English translation itself, and the other is to conduct research on the application of big data mining in the evaluation of translation discourse.

**Research findings:**

The research data show that the smallest gap between the sentiment orientation of the discourse evaluation perspective is the output of the language discourse, and the smallest gap in the attributes of the evaluation object is at the phrase level, and the gap value is 3.5; for the evaluation object, the maximum difference is 3.4.

**Research implications:**

With the development of science and technology and the economy, the public health environment has become more and more complex, and business English translation has received more and more attention. The sentiment analysis of evaluation discourse in this field is a means of expressing language characteristics. In order to enrich research in this field, the study of this article is necessary.

**Practical implications:**

This study has a deeper understanding of the affective analysis of evaluation discourse in public health environment business English translation. The clustering algorithm of big data mining technology applied can provide an important guarantee for the actual conclusion of this research and quantitative analysis of the positive evaluation and criticism of evaluation. To solve the various problems encountered in translation, so as to improve the translator's own translation level, and promote the research of translation methods in Chinese translation.

## Introduction

In recent years, China's economic strength and comprehensive national strength have steadily increased, playing an increasingly important role in the governance of global public health security. As the widely used academic language in the world, English is frequently used as the international carrier of the public health profession. Good command of English in the public health profession will undoubtedly help to strengthen the exchange of high-level research results in this field.

Business English is an important category of English, so the research of business English should also follow the English theoretical framework for important purposes (1). With the development of international trade, trade disputes are also increasing. At present, domestic and foreign scholars mostly analyze trade disputes from the aspects of politics, economy, law, and culture, but seldom do research on the aspects of the intervention methods of the two parties in the dispute and the language intervention resources (2). Thus, especially in China, only a few studies have paid attention to the communication strategies used by speakers in ELF communication in the business field. Business English has a wide range of social uses. In this process, people have formed some language habits in business communication. Linguistic research has also entered the commercial field, mainly the study of the contradictions that cannot be resolved by the unclear language in business letters and their translation procedures. The function of fuzzy language is to improve the accuracy of language expression, improve the efficiency of language expression, and realize the speaker's business self-statement (3).

The rapid development of data mining technology benefits from the existing big data sources in the world and the huge demand for transforming these data sources into information and knowledge sources (4). At present, the new technology of data mining has been recognized by society, and its application scope is not limited to a small range. Many companies with large amounts of data have also determined that they need to mine or predict some valuable new information from existing data (5). The concepts and rules involved in different companies are very different, so new requirements are put forward for data mining technology. Compared with the traditional synonym recognition method, the corpus provides a new perspective. Under a large number of intuitive and reliable situations, it can discover the different uses of synonyms in many aspects, making the recognition of similar words more scientific and comprehensive (6). With the rapid development of international trade, business English business terms are used more and more frequently on business occasions, and English translation training is a suitable topic for in-depth training (7). Most of them mainly study the Chinese translation of business English texts from the perspectives of functional equivalence theory, communicative translation theory, and functional skopos theory, although the theory has been used to study the Chinese translation of literary works relatively more.

The development of big data analysis technology has changed many areas of life, from information search to marketing. However, the use of data and text mining to understand the writing process in a language learning environment has not been fully explored to a great extent. Yim SM synthesizes the current methodology of collaborative writing and discusses how the new text mining tool can improve research ability (8). Taking commercial texts as an example, Novikova AV considers the practical aspects of meaning distortion when translating from one language to another in available machine translation (MT) systems and its basic method based on word-for-word translation. On the basis of analyzing the semantic and morphological features of actual text content and revealing the axiological and cognitive semantic features of subjective modality, a comprehensive functional method for translating business text is proposed (9). Abdi A proposes a method based on deep learning to classify user opinions expressed in comments (called rnsa). One of the unified feature sets represents word embedding, emotional knowledge, emotional transfer rules, statistics, and language knowledge, which has not been thoroughly studied for emotional analysis. Rnsa uses the cyclic neural network (Finn) composed of long-term and short-term memory (LSTM) to overcome several defects related to word order and information disappearance in traditional methods by taking advantage of sequential processing (10). Due to the availability of large amounts of data and the development of software tools to process them, the use of language resources beyond the scope of language research (e.g., commercial purposes) has become commonplace. Emotion analysis provides an interesting view of these data. It attempts to identify not only the polarity of the text but can also pursue further and more challenging goals, such as automatically identifying the specific entities and aspects being discussed in evaluative speech acts and the polarity associated with them. Moreno-Ortiz A studied the methodological aspects involved in creating such annotation patterns, motivated by the scarcity of information found in the literature (11). J indlerová analyzes a valence structure difference between the Czech English bilingual valence dictionary and the parallel syntactic annotation tree database, which represents the problem of the alignment of valence structures in the parallel corpus and the bilingual valence dictionary. Because of the dual possible distribution of semantic roles to the position of syntactic subjects, the dual interpretation of deep syntax can explain the role of actors. As a result, there are two different interpretations, one is a proxy and the other is a non-proxy, which conflicts with the data of syntax annotation (12). Shorabek A comprehensively describes the evaluative nature of metaphor in modern English. This paper determines the evaluation criteria of metaphor evaluability, considers the variation of metaphor semantic structure as the evaluation object and the agent as the evaluation donor, and distinguishes the axiological types, means, and methods of metaphor vocabulary and semantic group. The conditions for changing the evaluability of metaphor are systematic. The particularity of evaluability as a component of meaning in metaphorical semantics is determined (13). Fernández-Gavilanes M proposed a new method to predict emotion in online text messages (such as tweets and comments). This method is based on the text-based customization method based on unconventional trust analysis. This method uses a variety of mother tongue processing technologies and emotional features derived from the emotion dictionary. These dictionaries are created by a semi-automatic polarity extension algorithm to improve the accuracy of specific application fields (14). The research of business English translation has always attracted much attention. It is an inevitable development trend to apply big data mining technology to this field and make achievements. The above studies only focus on the application of data mining to language and language itself. The research on the combination of the two is relatively insufficient. In particular, there is little involved in the emotional analysis of evaluative discourse.

This study adopts the power research methods of comprehensive analysis, induction, and extraction, and combines scientific information with case analysis. In the part of the literature review, this paper mainly discusses the recent relevant research and the results of the feasibility study of translation and points out the problems of the current research. By referring to the definitions of relevant communication strategies made in previous studies, this paper marks the communication strategies in the corpus, uses the text image template, combined with the integrated data, outputs the evaluation objects and evaluation features, respectively, and calculates the sensitivity of evaluation features after the development of evaluation methods, so as to improve the evaluation features accurately. Quantitative analysis is used to analyze the distribution of intervention resources and the frequency of intervention resources in the corpus. Through quantitative analysis, it can help the audience or readers to more clearly grasp the position, viewpoint, and attitude of the speech subject.

## Big data and evaluation discourse of business English translation

### Business English translation

The rigor of business activities determines the strictness and accuracy of the carrier language. After World War II, science, technology, and the economy expanded rapidly all over the world (15). This expansion has increased the business contacts and proximity of members around the world. Different languages and cultures in different countries need intermediate support to express and exchange people's ideas, practices, and attitudes in science, technology, and technological development from all over the world, economic exchange systems, and promotion of research results. People of different nationalities and languages can have better communication and trade due to the existence of a common language. Therefore, business English has developed slowly due to the rise of global trade (16). In today's world, with the acceleration of global economic integration and the increasing importance of international trade in the global economy, trade English has become more and more important. With the development of the English business system, scholars have summarized the establishment and development of business English and conducted a lot of research on this basis (17). Language is a bridge of communication. English, as an international language, plays an important role in international trade. Business English translation has attracted more and more attention in trade activities. Accurate business English translation is not only conducive to the smooth development of trade activities but also conducive to reducing trade risks. For the above reasons, more and more experts and scholars have begun to study the Chinese translation of business English and the English translation of business Chinese. The translation of Intangible Cultural Heritage (ICH) terms is an important practical aspect of cross-lingual expressions related to ICH knowledge. Chinese ICH terms are heavily loaded with specific historical and cultural knowledge and regional characteristics (18).

People process language through advanced brainstorming activities. Although scientists have done a lot of research and achieved a lot of success, people still know very little about the basic knowledge of their own language (19). In fact, business English teaching has undergone years of development, from initial vocabulary teaching to positioning learning, and then to various efficient and interesting learning strategies. Initially, it was difficult for scientists to distinguish between Business English and General English. At that time, the use of professional vocabulary was to distinguish the characteristics of business English and general English, and business English was also an important point in the study of word selection (20).

### Emotional analysis of evaluation discourse

**Figure 1:**
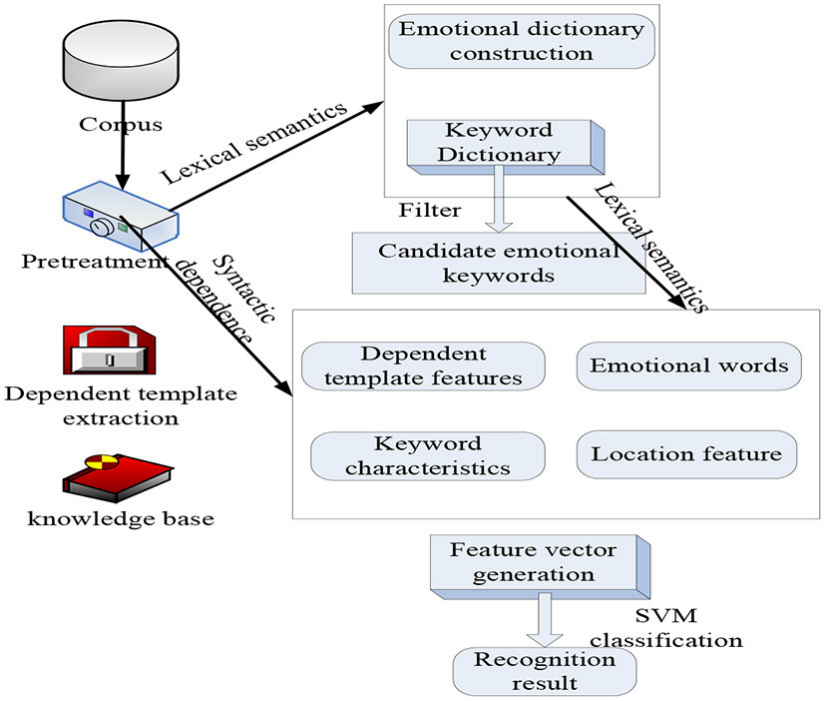
Data mining and emotional keywords.

Words cannot only be used to express information, exchange ideas, express feelings, and express social concerns, but more importantly, they can build a society together through communication (21). As a social practice, communication penetrates all levels of society, cultivates abilities and knowledge, and develops with the development of society, which can promote social renewal and social change. Critical discourse analysis and active discourse analysis are two perspectives of current discourse analysis. Active interactive analysis, as a new form of interactive analysis, actively looks at the construction of social interaction, advocates active response and problem solving, and makes up for the lack of critical discourse analysis that only pays attention to the unequal power relations in the text. Evaluation object recognition refers to the analysis and extraction of the object to which the reviewer expresses emotion in a review text, which is expressed as an entity in the opinion sentence that has a certain dependency relationship with the emotional word in the syntactic structure. It is inevitable for the development of the evaluation discourse field to fully explore the semantic connection between the evaluation object and the evaluation word, optimize the traditional model, and extract the evaluation object with wider coverage and higher accuracy. Appraisal theory is a supplement and expansion of the interpersonal meta-function proposed by Halliday in systemic functional grammar. It is a new method of studying the interpersonal meaning of the text. Fine-grained sentiment analysis no longer uses sentences as the unit to determine sentiment orientation but uses the topics or evaluation objects contained in the subjective text as the unit to determine their respective sentiment orientations. Emotional keywords are also called keyword phrases, which are composed of two elements: the keyword subject and the emotional subject. Topic keywords are used to summarize chapter topics, and emotional topics are used to summarize emotional feelings. It is mainly used to analyze and study the behaviors, concepts, and conditions presented by many evaluation sources in the text. The differences in different fields have caused many words to have different translation methods in different fields. We assume that the translation system has received the same corpus training at the same point; then, the translation system becomes more effective in interpreting data at the same point, while translating other fields. With the development of statistical analysis and machine learning technology, corpus scientific analysis has become the first. Traditional sentiment analysis technology has a large amount of analysis scale and accuracy and cannot meet the needs of the ever-increasing Internet users (22). Taking into account people's expression habits, according to the different ways of emotional expression of commenters at the beginning, middle, and end of paragraphs, the importance of sentences is defined by the statistical data of the training corpus, and then the sentiment of paragraphs is predicted by sentence sentiment. Because sentiment analysis often focuses on sentimental words containing emotions, behaviors, and perceptions, the automatic separation of sentimental words and verbal words can promote follow-up actions. The meaning of the sentiment dictionary is to synthesize part of the seed sentiment words first and then expand the seed words through technology to complete the construction of the sentiment dictionary. Machine learning classification models, such as support vector machines, maximum entropy, and conditional random fields, are used to complete the sentimental judgment of sentences. From the perspective of the size of the role, psychoanalysis and sound analysis can be divided into sub-level and sentence level. Currently, it is generally effective to reach the sentence level (23). Therefore, how to use fictitious analysis technology to allow computers to automate and analyze network information, provide decision-makers with more shared information and decision-making information, help companies understand consumer habits, and help users fully understand product information has become a development trend. Figure 1 shows the construction model of sentiment analysis of evaluation discourse in business English translation. Since emotional words and keywords are the two main components of emotional keywords, paper definition expansion and word-building structures are used to capture the meaning of words (24). Data are the basis for the success or failure of mining work. Therefore, in the problem definition stage, it is necessary to further understand the data, such as determining the specific data required for data mining, describing the data, and checking the quality of the data.

Generally speaking, the application of data mining in vocabulary is divided into training samples and test samples, and then the training model is applied to the test words to compare the isolation effect of the isolation model, and finally, the best model is selected to complete the emotional isolation of the text.

### Application of big data mining in language

In the process of big data mining, different themes should be formulated for different users. The topic is a standard for classifying data at a higher level, and each topic corresponds to a macro-analysis field, that is, integrating different topics according to certain standards and transforming the original data structure from application-oriented to theme-oriented. The huge potential value of big data prompted the emergence of big data mining technology (25). Compared with software tools and advanced data management techniques, the functions of human data analysis tools cannot provide decision-makers with the necessary knowledge required for decision support, so there is a unique formula: rich data, knowledge (26). Traditional data mining algorithms face many challenges when processing big data. Improving the original data mining algorithms and combining cloud computing technology is an effective way to deal with big data mining. Cloud computing provides strong technical support for big data mining in terms of computing and storage. Therefore, the combination of cloud computing technology and big data research is an inevitable trend for big data mining research and has important practical significance. Convert the search conditions into the target language and then search in the same language. The processing flow is the first step to obtain the retrieval conditions to be processed, then preprocess the retrieval conditions to obtain keywords, then perform translation through the dictionary to obtain the same keywords as the target language, and finally perform searches in the same language. At the same time, it is necessary to solve data consistency problems, such as data matching, numerical conflict, and data redundancy involving multiple data sources, as well as the need to convert the original data into a form suitable for mining. Therefore, in the data preprocessing stage, data cleaning, data integration, and transformation are required to prepare for further analysis. The application of big data mining in language vocabulary and emotional color is shown in Figure 1.

In the task of word translation mining, this paper improves the method based on distributed representation and proposes a method based on a neural network. First, the words are clustered, and then the neural network is used to replace the linear function in each category to mine the mapping relationship between the word vector representations. The neural network is used instead of simple linear mapping to enhance the accuracy of mapping between words in different languages. It also performs clustering operations on words to enhance the association information between words in the same language. All operations are performed on a monolingual corpus. After the improvement, it can better mine the association between words in the same language and the mapping relationship between words in different languages, so as to achieve the purpose of improving the accuracy of word mining. Big data is used to analyze language sensitivity, especially business English language assessment. First, data mining clustering algorithms can be applied. Positive discourse and critical discourse are two categories. The calculation formula based on their basic emotional characteristics can be expressed as follows:


(1) $$\begin{array}{l} \eta_{i}=\frac{1}{\left||A_{i}|\right|}\underset{P_{h}\in A_{i}}{\sum }P_{h},p=\left\{p_{1},...,p_{h}\right\} \end{array}$$

(2) $$G=\overset{n}{\underset{i=1}{\sum }}{\sum^{\text{​}}}_{p_{h}\in A_{i}}(P_{h}-\eta_{i}{)}^{2}$$

G represents the clustering objective function, that is, in the data mining process, the computer system processes keywords according to the language according to the clustering objective function to classify emotional discourse; n represents the nth data node; *P*_*h*_ represents the positive utterance; and *A*_*i*_ represents the overall input data. Phase-specificity was used for refinement, as follows:


(3) $$\begin{array}{l} F_{p_{h}}\left(d\right)=|P_{hf}-P_{if}|/\left(\max_{f}-\min_{f}\right) \end{array}$$

(4) $$\begin{array}{l} P_{A_{i},p_{h}}=\left\lfloor \left|P_{if}-P_{hf}\right|\right\rfloor /\frac{1}{E_{f}-1} \end{array}$$

(5) $$f(A_{i},P_{h})=\{\begin{array}{cc} 1 & P_{if}=P_{hf}\\ 0 & P_{if}\neq P_{hf} \end{array}$$

*F*_*P*_*h*__(*d*) represents the degree of dissimilarity between the data point *P*_*h*_ and the d-th attribute of the data point *P*_*f*_. *P*_*A*_*i*_, *P*_*h*__ represents the attribute of an ordinal set, which is one of the dissimilarities of discrete attributes. There is no difference between the discourse of different emotions in other dimensions, that is, it is described by *f*(*A*_*i*_, *P*_*h*_). After categorizing the data and combining the calculations, this process can be expressed as follows:


(6) $$\begin{array}{l} C_{i}=\frac{1}{\overset{n}{\underset{j-1}{\sum }}C_{i}^{j}}\overset{n}{\underset{j=1}{\sum }}1SUM \end{array}$$

SUM represents the summation function, and *C*_*i*_ represents the central characteristic of the i-th partition. J represents the number of data nodes. The distributed data processing process is expressed as follows:


(7) $$\begin{array}{l} E\left(A=a\right)=\frac{1}{o}\sum_{j}\delta_{j}\left(a\left\{j\right\}\right) \end{array}$$

(8) $$\begin{array}{l} E\left(a\right)=\frac{1}{i}\sqrt[{i}]{\delta_{i}}{\left(a\left\{i\right\}\right)}^{ij} \end{array}$$

A similar group has not only common syllables but also its own unique syllables. The definition is roughly the same, but the method is different. These data are data with no direction, but a certain central characteristic is selected for planning and display. *E*(*a*) represents an exponential function, and O represents a normalization factor. δ_*j*_ represents the number of instantiations of the criterion. The support of emotional words in the database can be calculated using formulas:


(9) $$\begin{array}{l} Support\left(i\right)=SUM{\left(A\right)}_{ij}/\left(n-ij\right) \end{array}$$

(10) $$\begin{array}{l} B\left(a↔b\right)=a\cup b/\underset{i=1}{\sum }\left(j-1\right) \end{array}$$

*B*(*a*↔*b*) is a data node greater than or equal to the lowest confidence level, which also belongs to the scope of meaningful rules. The calculation of the joint probability of a single word in the joint distribution can be expressed as follows:


(11) $$\begin{array}{l} \alpha_{i}\left(a\right)=\sqrt{\frac{\partial }{\partial \beta_{i}}}\log F_{\beta }\left(A=a\right) \end{array}$$

(12) $$\begin{array}{l} \beta -\partial =-\underset{a^{{}^{\prime }}}{\sum }F_{\beta }\left(A=a^{{}^{\prime }}\right)l_{i}a^{{}^{\prime }} \end{array}$$

(13) $$\begin{array}{l} f^{+}\left(a_{i},a_{j}\right)=1/\beta_{ij}^{+} \end{array}$$

α represents the set of node vectors, and β is the variable corresponding to the node. *f*^+^is 0 to 1-dimensional information attribute. Apply it to the stochastic gradient to decompose, and then can be expressed as follows:


(14) $$\begin{array}{l} E=\frac{1}{2}{\left(u-\beta^{t}\varepsilon \left(a\right)\right)}^{2} \end{array}$$

(15) $$\begin{array}{l} \beta \leftarrow \beta +\varpi_{t}\left(v_{t}-\beta_{t}\left(a_{t}\right)\right)\leq 0 \end{array}$$

(16) $$\begin{array}{l} \beta_{i}•\leftarrow \beta_{j}+\frac{1}{ij•}\left(t-\beta_{i}\right)\left\{0,a\right\} \end{array}$$

The initial number of each category is ε, and the convergence rate β and convergence β_*i*_ of the learning rate β_*t*_ have relatively large fluctuations on the algorithm, and u is the parameter. Rule prediction for some vague or polysemous words can be calculated as follows:


(17) $$\begin{array}{l} R_{t}/R_{c}=\frac{r_{2}}{r_{1}}⊗\frac{r_{3}}{r_{4}}⊗\frac{r_{2}}{r_{1}}... \end{array}$$

(18) $$\begin{array}{l} R\left(f_{1}|f_{2},t,c\right)=R\left(f_{1}|f_{2},Z_{t,c}\right) \end{array}$$

*R*_*t*_, *R*_*c*_ represents the accuracy rate and recall rate, respectively, *r*_1_ is the total number of predictions, and *r*_2_ is the number of accurate ones. *r*_4_ is the actual number of *r*_3_ predicted occurrences, f is a variable, and z is the rules. The predicted probability can be given as follows:


(19) $$\begin{array}{l} w_{tc}^{{}^{\prime }}=\overset{r_{i}}{\underset{i=1}{\sum }}W_{itc},W_{tc}\left(t-c\right) \end{array}$$

(20) $$\begin{array}{l} W_{i}\left(r_{1}=male,r_{2}=low,r_{2}=low,r_{4}=low,r_{3}=no\right) \end{array}$$

W represents the distribution index coefficient, and $w_{tc}^{{}^{\prime }}$ represents the set of coefficients to be classified.

## Experiments on big data analysis of discourse sentiment

### Business English translation

Business English is based on ordinary English. It has the linguistic features of ordinary English and is a combination of business knowledge and ordinary English. Therefore, business English has its inherent uniqueness. Findings show that teachers are positive about the appropriateness of applying the framework in the context of 21st-century skills and a viable degree of effectiveness in a learning environment. Participant teachers who applied this approach using the conceptualized paradigm in teaching Business English recommend its efficacy due to relevant pedagogical implications and practical principles that were discovered (27).

**Figure 2:**
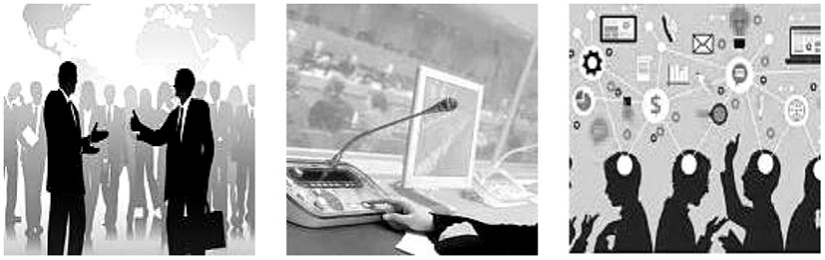
Business English, translation, and business.

English e-commerce has developed into universal and useful culture, and it is imperative to learn language characteristics and translation skills. With the rapid development of the Internet, companies are facing major changes in their business models. Among them, the use of commercial websites is the most obvious, as shown in Figure 2. E-commerce is evolving into a popular business model. Whether companies can make full use of the Internet is the key to their survival.

For texts of the same type, such as business texts, the usage of certain terms and sentence patterns is similar or even consistent. As mentioned above, the translator refers to the concept of the intertextuality of genres and has come to the conclusion that the translation of certain fixed sentence patterns can be used repeatedly as symbols of special meaning in different business texts.

**Figure 3:**
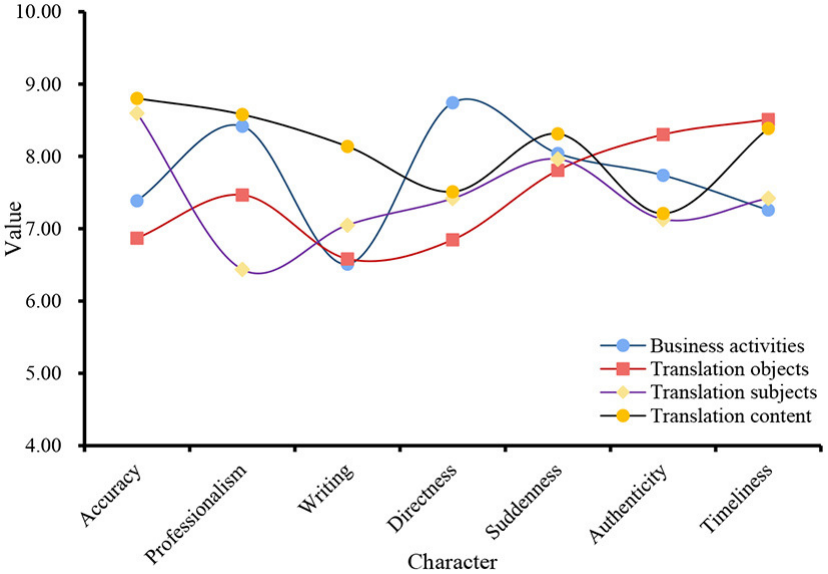
Features of business English translation.

The translation of English e-commerce requires not only a strict language foundation but also familiarity with translation skills because English business translation has its unique characteristics. Understanding the characteristics and translation skills of English e-commerce is an important and clear definition for improving the efficiency of English e-commerce dictionaries and improving the quality of translation. The characteristics of business English translation include accuracy, professionalism, writing, directness, suddenness, truthfulness, and timeliness. When translating e-commerce English vocabulary, the characteristics of vocabulary, translation rules, and e-commerce knowledge should be organically and closely linked, so as to accurately translate e-commerce English words and achieve the best translation and communication effect. For this reason, this article acquires data from four aspects: business activities, translation objects, translation subjects, and translation content. The relationship between English translation characteristics and these four aspects is shown in Figure 3.

**Figure 4:**
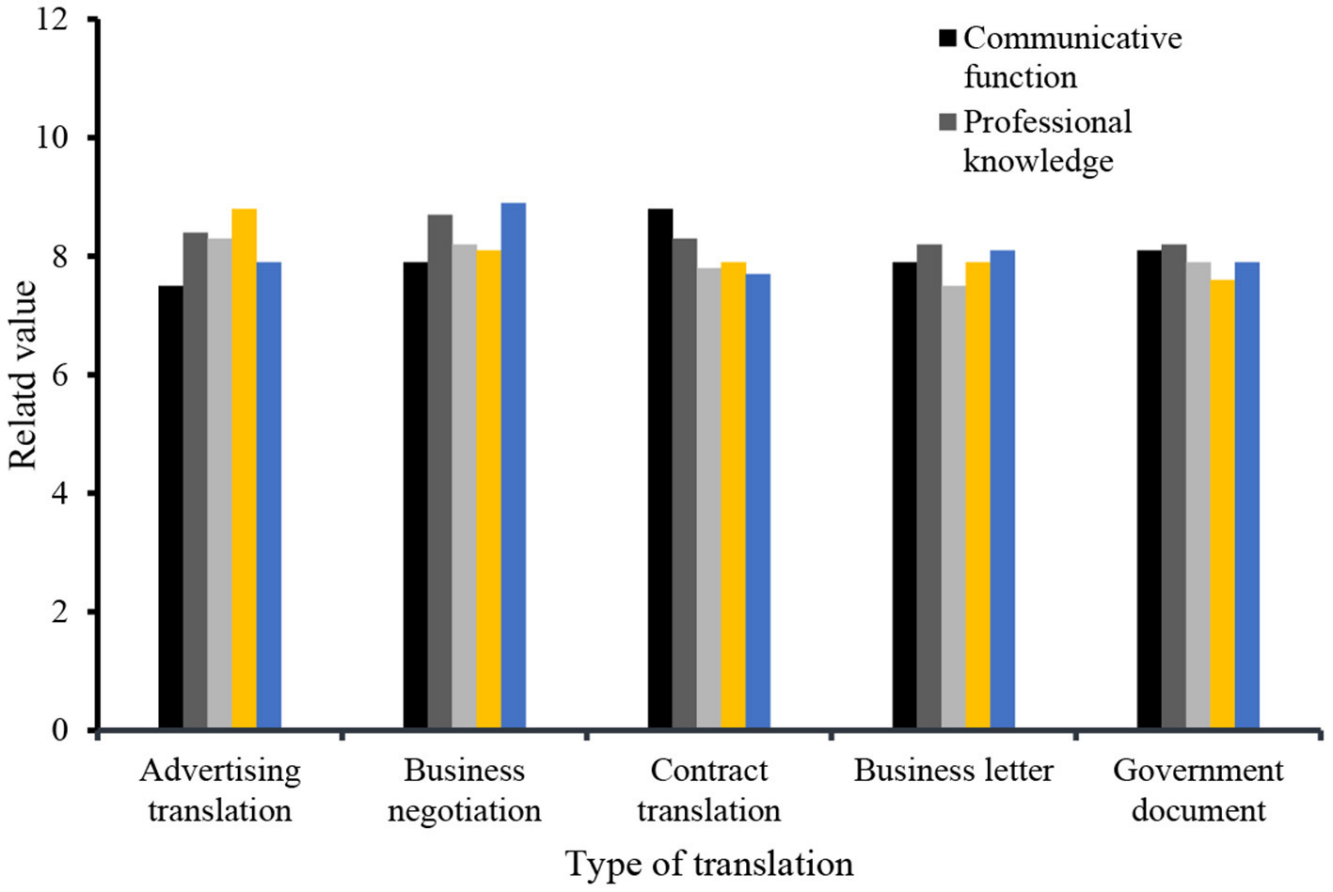
The specific content of business English translation.

E-commerce English is a practical style with many language features. Related studies believe that the source language interference phenomenon in Chinese-English translation is mainly manifested at the vocabulary level, syntactic level, stylistic level, and cultural level. The fundamental causes of source language interference mainly include four aspects: differences in Chinese-English vocabulary collocation, differences in Chinese and English syntax, differences in cohesive methods and structures of Chinese and English texts, and differences in cultural background and language environment. This study covers the compatibility between business English translation, content advertisement translation, business negotiation, contract translation, business correspondence, communication between government documents and translators, professional research, language skills, cultural background, and management skills. English translation is the main way for both parties to understand each other and enhance trust. Therefore, it is necessary to have a clearer logic in translation and to clearly express the wishes of both parties, as shown in Figure 4.

Business English translation includes the translation of business letters, translation of government documents, translation of commercial advertisements, translation of travel texts, translation of product brochures, translation of corporate profiles, and translation of business contracts. The translation of different contents has a certain format.

Based on the development of more complex matching algorithms, adding rules to the relationship between adjacent terms improves the division effect to a certain extent. Currently, a manually segmented corpus is commonly used for model training, and the model is annotated and coded to complete the segmentation. However, the construction of corpora in different fields requires a lot of manpower and material resources, and the construction of partition training is a trivial matter.

### Big data processing data algorithm application

With the advent of the era of big data, the world is facing unprecedented information retrieval challenges, and Internet information technology is also accelerating. The Internet is a driving force to promote social unity, stability, and system development. It is essential to the ability to accurately, timely, and effectively capture short text emotional information. However, business English translation has the characteristics of freedom, flexibility, and lack of standardization, and emotional information is affected by time, space, region, and emotional events caused by characters. In machine learning methods, emotion definition as feature selection is to use emotion words as feature functions in training evaluation. The specific task is to insert the sentiment dictionary into the custom dictionary of the partition system and set a special selection command that matches the dictionary definition, which is simple and clear. In addition, the feature selection process and word segmentation process can be carried out at the same time, which is simple to implement, low in complexity, fast in processing speed, and can also greatly reduce the dimensionality reduction calculation work for text. The method based on a dictionary or basic knowledge is to first compile the words or technical solutions in the dictionary into the first dictionary, and then use a method to expand the area of the dictionary. The main role of big data mining in business English translation is to obtain information, such as data characteristics of short text analysis and mining data. The main task is to obtain a large amount of English vocabulary data from the Internet and use a specific format for information storage, then text partition and information display of the received communication information, and finally determine the rating and emotion type. First, we analyze the reliability of big data mining algorithms, as shown in Table 1.

**Table 1:** Reliability analysis of big data mining algorithms.

	**Text**	**Vocabulary**	**Sentence**
Decision tree algorithm	0.56	0.66	0.57
Collaborative filtering	0.67	0.68	0.62
Matrix factorization	0.64	0.68	0.65
Clustering Algorithm	0.77	0.74	0.79

As can be seen from the above table, we mainly conducted research around four algorithms for big data mining. On the whole, the clustering algorithm is more accurate and reliable than the other three algorithms in processing text, vocabulary, or sentences. For this reason, we analyze the overall situation of the big data mining clustering algorithm, and carry out data statistics for the content of business English translation, as shown in Table 2.

**Table 2:** Investigation of the content of business English translation by the clustering algorithm.

**Clustering algorithm**	**Culture**	**Economy**	**Education**	**Healthy**
Traditional algorithm	0.526	0.608	0.621	0.687
Improve algorithm	0.743	0.772	0.748	0.801
This research	0.741	0.802	0.673	0.812

Table 2 mainly analyzes a specific situation around the clustering algorithm in business English translation content, such as culture, economy, education, and health. From the traditional algorithm and the improved algorithm, as well as the improved algorithm of this research, the evaluation shows an overall optimization. The situation also proves the important value of the research in this article and the credibility of the data. Traditional algorithms can achieve accurate word segmentation, but their ability to find new words is poor; advanced algorithms are effective in finding new words and isolating regular words, but require a large amount of simulated corpus as a training tool. Sometimes, a marked corpus will merge with human thinking factors, leading to word segmentation errors.

**Figure 5:**
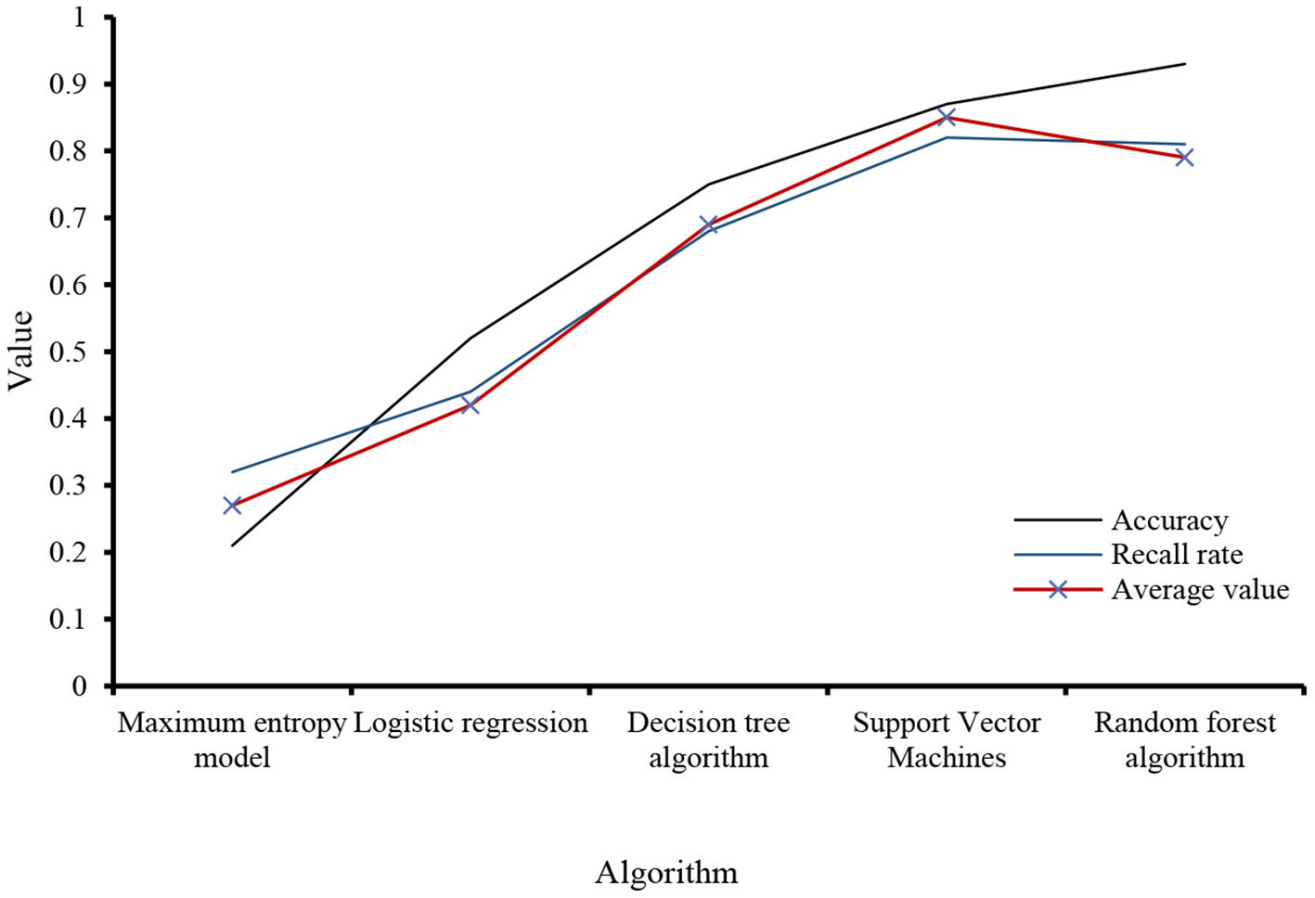
Algorithm performance.

It is relatively easy to determine the polarity of emotional words, but it is difficult to quantify their polarity strength. This is due to the characteristics of the fuzzy semantics of the Chinese language and the subjectivity of the review text. Specifically, the polarity strength of Chinese emotional words is vague. Use integral analysis to obtain a set of objects to evaluate competitors, and then use the SVM model and the scale model to achieve structured sound extraction. This task is divided into three steps: (1) text editing, including coverage information and text sharing; (2) writing translation text; and (3) evaluating evaluation factors in the candidate set. Analyze the performance of each algorithm adopted in this experiment, as shown in Figure 5.

E-commerce English texts have high requirements for logic. Therefore, attention should be paid to the connection between sentences in the translation process. It mainly focuses on comparing the accuracy rate and recall rate of algorithm data. If there are characteristic words in a sentence, the object of the sentence is always related to a meaning, which affects the characteristics of emotional words to a certain extent. Because people's attitudes, concepts, attitudes, etc., are very different, they are eager to express their appeals, and they are very different. Recorded the relevant data of the statistical examples of emotional characteristics in the discourse evaluation, as shown in Table 3.

**Table 3:** Statistical examples of emotional features in discourse evaluation.

**Emotion**	**Negative emotions**	**Positive emotion**	**Weights**
Phrases	307	39	0.07
Number of emotional words	473	97	0.21
Text	702	274	0.43
Translation feature level	542	227	0.29

**Figure 6:**
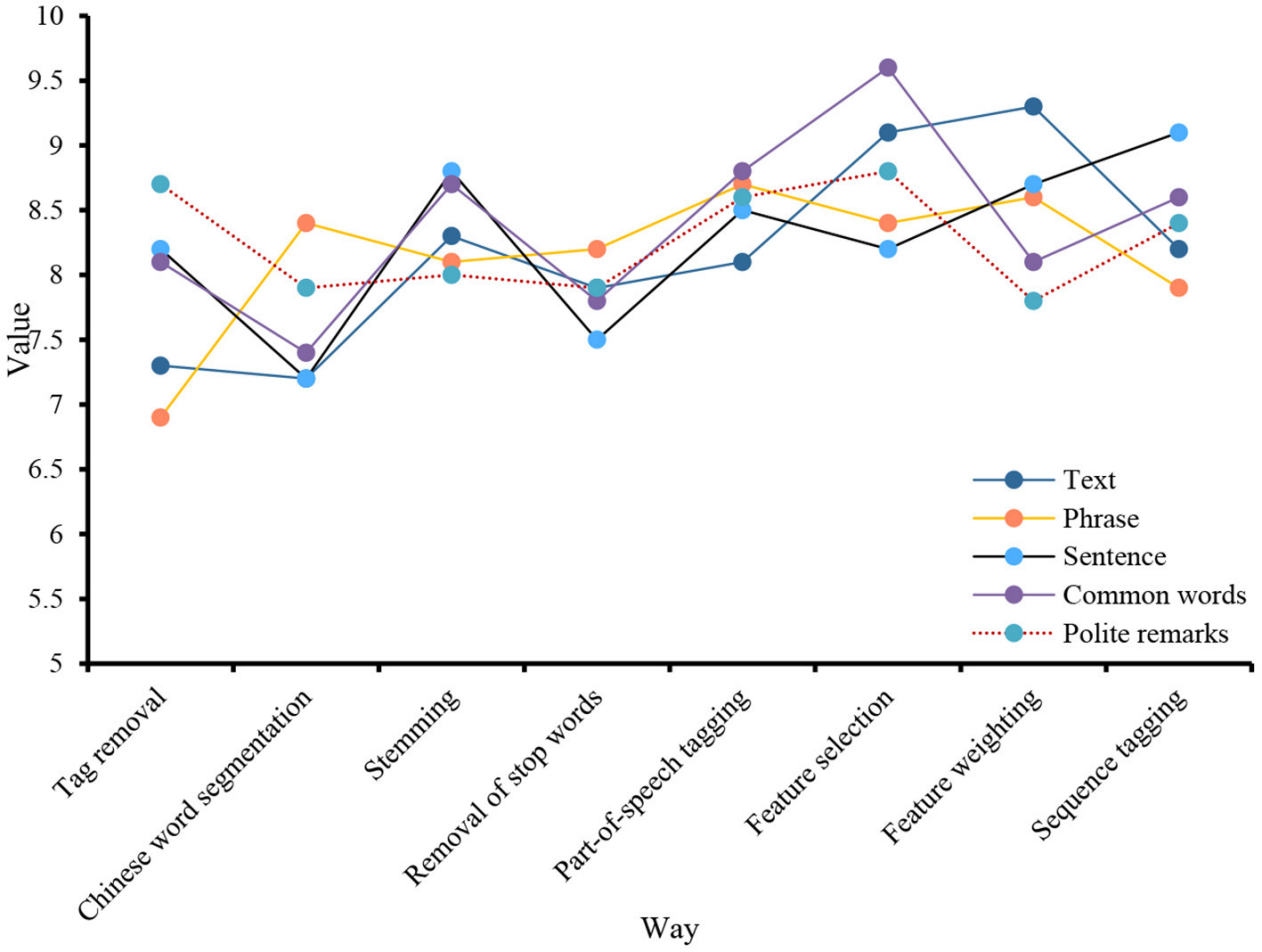
Translation process.

On the one hand, under the guidance of compliance, the reasons for business divergence are indicative. According to this principle, both the speaker and the listener are responsible for misunderstandings in business communication. If the speaker makes a mistake in understanding the source of the listener's business situation, it will present the listener with negative inaccuracies and weakly related issues, leading to inaccuracies, and thus, discourse misunderstandings. It continues to attract new achievements in science, psychology, and other social sciences and continues to promote the exploration of programming activities on social media. On the other hand, cognitive science focuses on the cognitive functions in the brains of language users. Therefore, many scholars believe that keyword analysis should lead to more cognitive science research results to help expand keyword analysis. Tag removal, stemming, etc., are all processes of text preprocessing before translation. Perform relevant data statistics on these processes, as shown in Figure 6.

In the process of translation, translators should use accurate words and clear concepts, especially the units and data. Compared with other forms of language expression, business English pays more attention to the accuracy and fidelity of the content. Only in this way can the entire translation be used. In the user interface, we merged the correct information and reduced the size of the information vector of the translation data, thereby improving the performance of subsequent data mining algorithms. Data processing is mainly to process data and express a data structure before data processing. The effective data processing and presentation of intelligent data structure will improve the statistical improvement of data mining and the accuracy of data mining results. Data processing is an important part of data mining. The principle of translation method holds that the clarity principle of business text language is supported by clearly conveying the message to be conveyed, avoiding ambiguity and ambiguity, and being concise and organized. Record the different characteristic values of the data, as shown in Table 4.

**Table 4:** Experimental result data of different eigenvalues.

	**Accuracy**	**Recall rate**	**Mean**
All eigenvalues	0.653	0.527	0.602
Translation length eigenvalue	0.721	0.537	0.584
Outlier	0.572	0.693	0.592
Emotional distribution outlier	0.587	0.601	0.638
Content similarity	0.662	0.583	0.571
Repeat times	0.682	0.591	0.593

**Figure 7:**
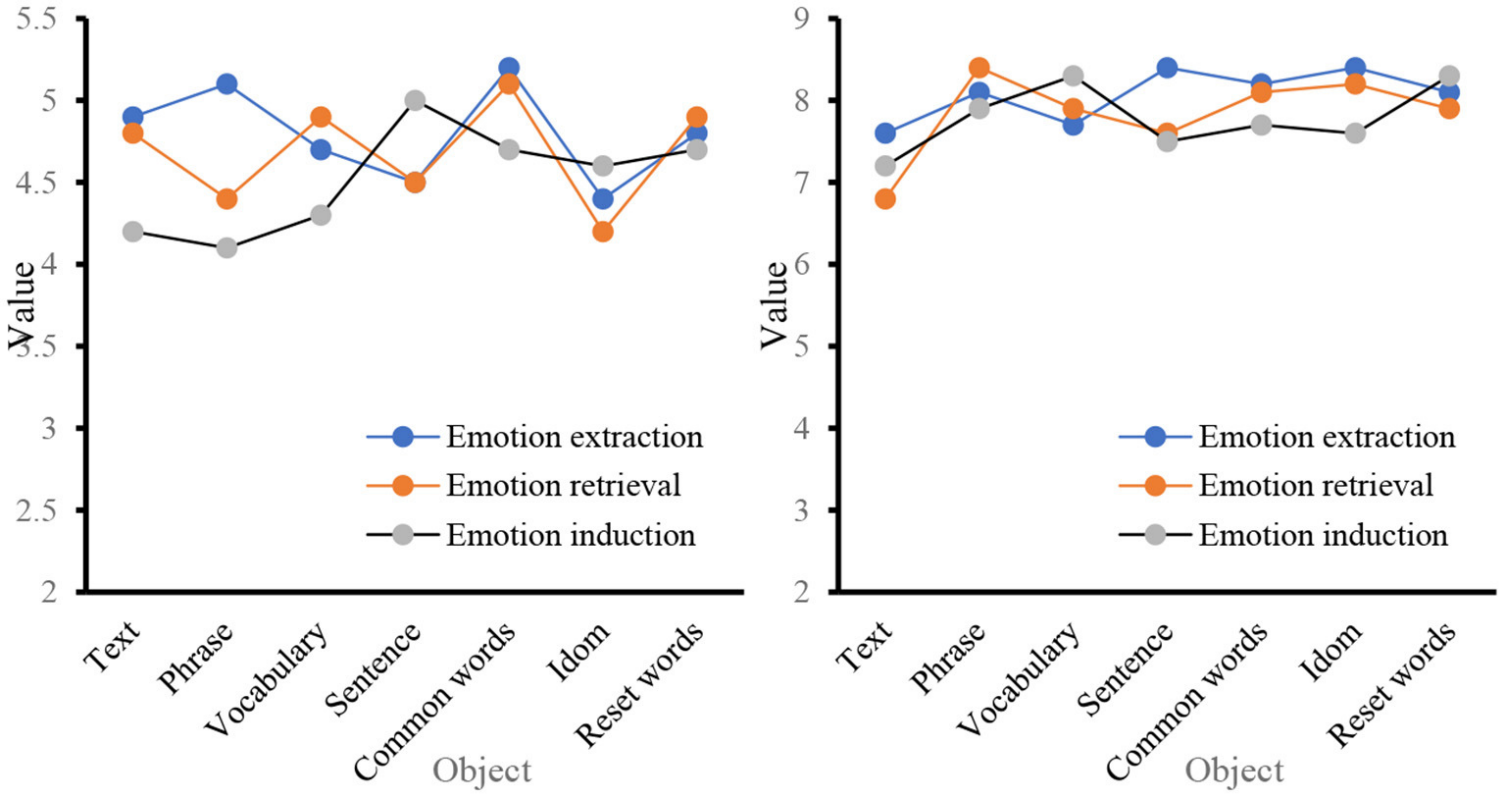
Length of evaluation discourse.

Based on the size of the translation evaluation discourse, the sentiment of the evaluation discourse is classified and analyzed. The data before and after the analysis are shown in Figure 7.

**Figure 8:**
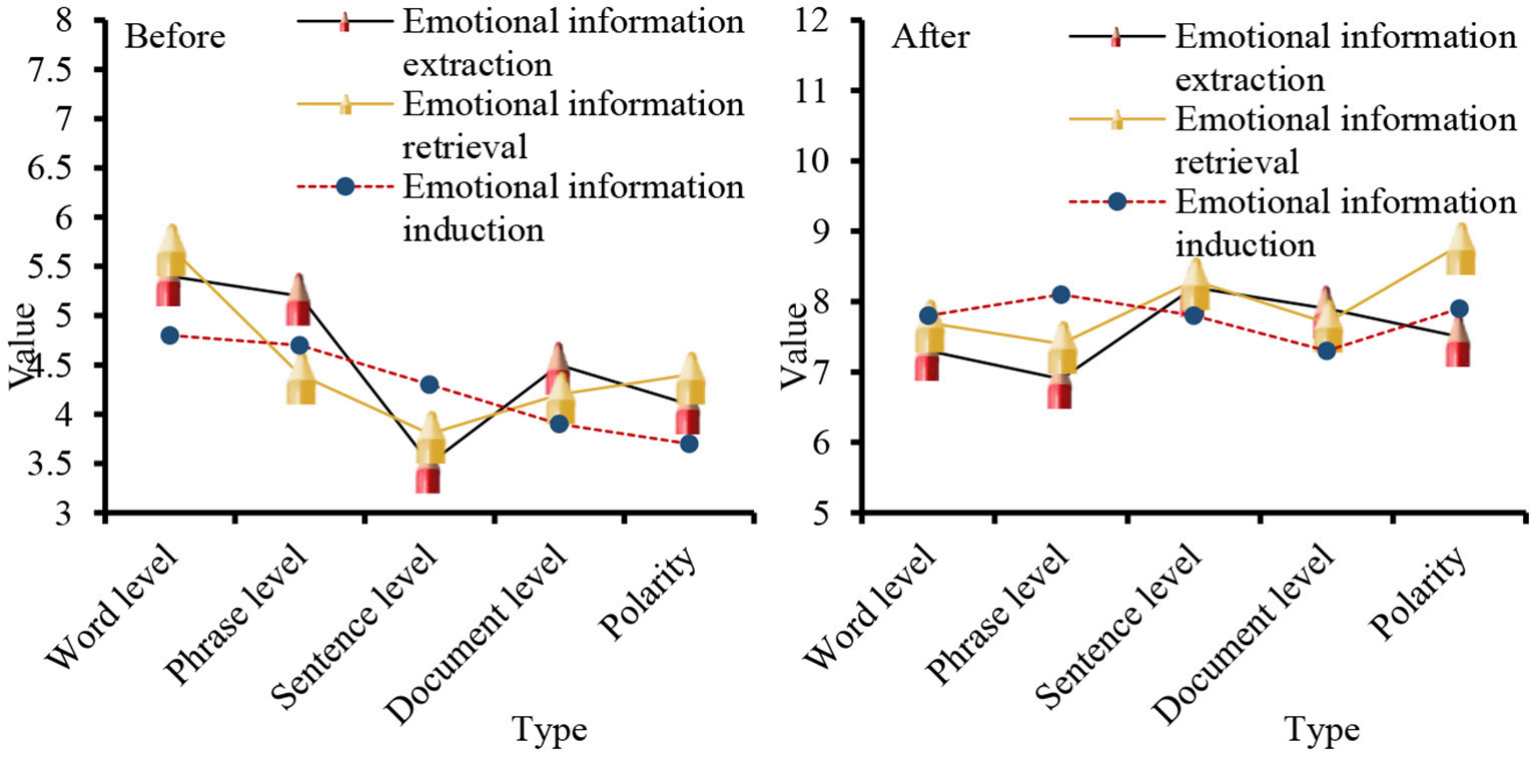
Classification task statistics.

In recent years, with the increasing development of text processing technology, the research scale of sentiment analysis has gradually expanded, and it is mainly applied to classification tasks, such as word level, phrase level, sentence level, document level, and commendatory and derogatory polarity. For the three aspects of emotional information extraction, emotional information search, and emotional information submission, the before and after application of big data mining in the emotion of business English translation is analyzed. The first is the data on the classification task. According to the existing knowledge structure and receptive ability of the main readers of the text, the information degree of the text should be adjusted appropriately, so as not to reduce the depth and interest of the text, but also to prevent readers from affecting their correctness of the text due to lack of knowledge, as shown in Figure 8.

**Figure 9:**
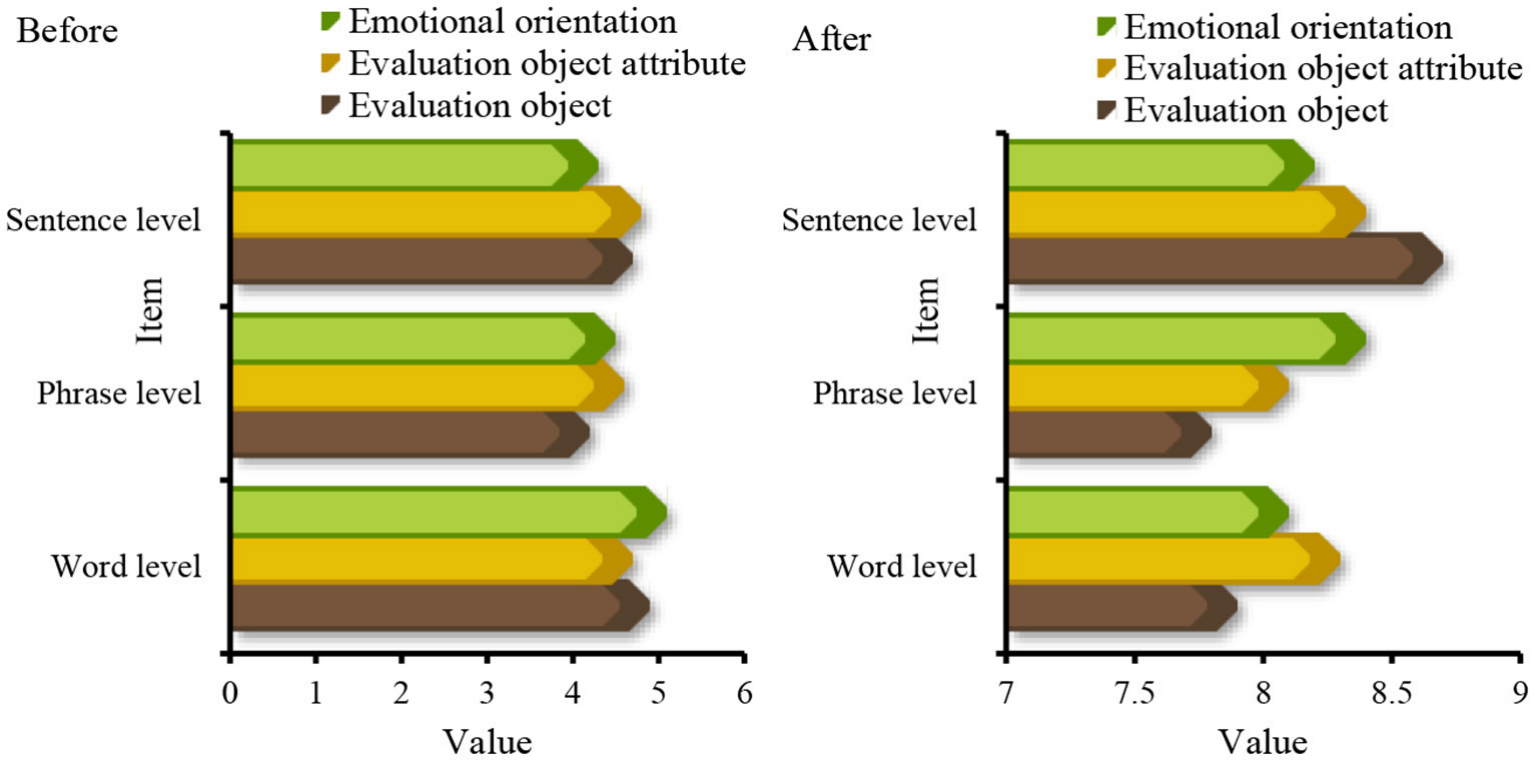
Sentiment analysis from the perspective of discourse evaluation.

In Figure 8 show that before the big data mining algorithm was applied to business English translation, the levels of emotion information extraction, emotion information search and emotion information submission in the word level and phrase level classification tasks were all lower than the level observed after the application, and the overall gap was relatively large. From the three aspects of the evaluation object, evaluation object attribute, and emotional orientation, a data record is made for the three aspects of emotional information extraction, emotional information retrieval, and emotional information induction, as shown in Figure 9.

**Figure 10:**
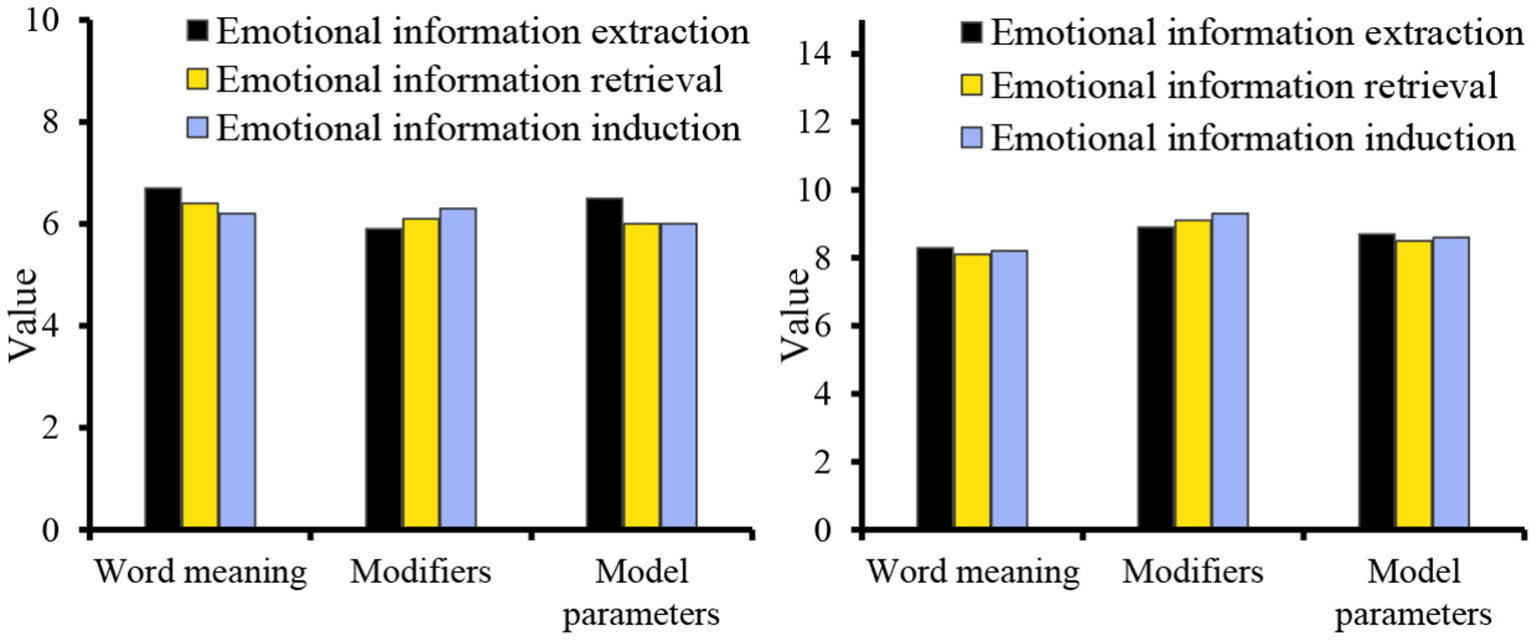
Sentiment analysis of the text.

From the perspective of discourse, its intentionality determines the choice of translation methods and the processing of translations in the process of translation, aiming at the coherence of discourse to achieve the purpose of communication. The data analyzed from the perspective of discourse evaluation Figure 8 shows that in the word level, phrase level and language discourse output before and after the application of data mining algorithm, the gap between language discourse output is the smallest, and the smallest gap in the evaluation object attribute is 3.5; for the evaluation object, the maximum difference is 3.4. From the three parts of word meaning, modifiers, and model parameters, the three aspects of emotional information extraction, emotional information retrieval, and emotional information induction are studied. The data are shown in Figure 10.

When big data mining is applied to business English translation, the largest difference in word meaning is emotional information induction. The numerical difference of modifiers in emotional information extraction, emotional information retrieval, and emotional information induction is 3. In terms of the parameters of the model, the numerical difference in emotional information extraction is the smallest, which is 2.2. Sentiment analysis can be divided into several research levels, such as word-level, phrase-level, sentence-level, chapter-level, and multi-chapter-level.

## Discussion

The main research contents of word analysis are machine learning, data mining, probability process, linguistics, statistical analysis, and so on. The objective of this study has also attracted the attention of many scholars because this research method is to transform the discourse comment text of business English translation into feature vectors for detailed analysis, so the construction of feature vectors plays a certain role in the correctness of affective tendency analysis. The main feature of the model is composed of adjectives and modifiers. The dataset containing positive and negative comments is applied to the classifier support vector machine for classification. The conditional random field model is used to determine the emotion expressed by the local content in the text. Compared with the traditional machine learning method, this analysis method can analyze the emotion of the text more accurately at multiple levels, which provides great help for the follow-up research. By adding statistical indicators, such as part of speech distribution outlier and emotion distribution outlier in the model, false comments can be marked more effectively. The credibility of affective data is the key issue of the affective analysis task. It is the guarantee and basis of all later affective analysis research and application. Only by filtering out false affective information on the corpus, all subsequent affective analysis research work will be more meaningful. From these big data and Internet short texts that cannot be organized quickly, accurately, and efficiently, analyzing and retrieving useful emotional elements and emotional reasons has become the key to Internet text analysis and related application research. In order to facilitate text data mining, a common solution is to read the text frequency, write a text vector according to the vocabulary, and specify the characteristics of the object according to the frequency data in the vector. If the problem of mother tongue understanding is transformed into a problem of machine learning, the first step must be to find a way to digitize these symbols. Therefore, when translating English, it is necessary to combine the customs and cultural characteristics of different countries, and adopt scientific and reasonable translation methods to avoid improper translation, resulting in disputes and misunderstandings.

## Conclusion

In the current stage of rapid development of global public health, it is very important to build a high-level public health system and train innovative public health talents with an international vision. On the basis of refining the progress and achievements in the field of public health in China, improving the level of internationalization will undoubtedly enhance the world's understanding of the development of public health in China, improve the voice of public health, and provide Chinese wisdom for the development of global public health. The cultivation and improvement of English ability is the premise of education internationalization. Sentiment analysis is an important research topic in information mining technology. This research focuses on practical applications. The emotional impulse in the English translation business is affected by the emotional perception caused by time, space, environment, and characters. Behind the emotional problem is the word processing technology of information about the cause of the emotion. Compared with the progress of foreign research, the sentiment analysis of discourse evaluation in business English translation has been behind for several years, and due to the complexity and diversity of language and culture, it has become impossible to directly transplant foreign research results into Chinese text-related research programs. Therefore, there are still some challenges for scholars who study the sentiment analysis of discourse evaluation in business English translation, because there are few resources and research results, and we need to further explore and work hard. But at present, domestic scholars have made some achievements in the field of text sentiment analysis. To accurately translate vocabulary, one must understand the general knowledge and definition of the suitability of the relevant company, be good at using software and tools to find information, and be willing to spend time and energy to ensure that there are corresponding words in the vernacular. The clustering algorithm of big data mining is complicated, and it is necessary to manually set the cluster density and the expansion target between clusters during the execution process. The clustering results are greatly affected by the experimental dimensions. The research angle of this research is relatively single, and the follow-up work will conduct more comprehensive research on this basis. In this paper, by analyzing the text, because the translator is limited by his own professional knowledge and lack of experience, the analysis of the text is still not thorough enough. In addition, the translation methods discussed in the article are not exhaustive, and we hope to improve them in the future.

## Data availability statement

The original contributions presented in the study are included in the article/supplementary material, further inquiries can be directed to the corresponding author.

## Ethics statement

The studies were reviewed and approved by the Hunan University of Finance and Economics, Nanyang Technological University and Guangdong University of Finance and Economics. The participants provided their written informed consent to participate in this study.

## Author contributions

SL and YC contributed to the writing. KX performed data collection. JL contributed to data preprocessing. All authors contributed to the article and approved the submitted version.

## Conflict of interest

The authors declare that the research was conducted in the absence of any commercial or financial relationships that could be construed as a potential conflict of interest.

## Publisher's note

All claims expressed in this article are solely those of the authors and do not necessarily represent those of their affiliated organizations, or those of the publisher, the editors and the reviewers. Any product that may be evaluated in this article, or claim that may be made by its manufacturer, is not guaranteed or endorsed by the publisher.
